# IR Map of the Human Cell

**DOI:** 10.1021/acs.analchem.6c00513

**Published:** 2026-06-22

**Authors:** Anna Antolak, Aleksandra Pragnaca, Karolina Augustyniak, Syeda Takmeel Zahra, Łukasz Pięta, Adrianna Wislocka-Orlowska, Katarzyna Majzner, Malgorzata Baranska, Kamilla Malek

**Affiliations:** † Faculty of Chemistry, 37799 Jagiellonian University in Krakow, Gronostajowa 2, 30-387 Krakow, Poland; ‡ Doctoral School of Exact and Natural Sciences, Jagiellonian University in Krakow, Lojasiewicza 11, 30-348 Krakow, Poland

## Abstract

Classic infrared (IR) microscopy is limited by the diffraction limit, which obscures subcellular heterogeneity, and by the complex overlap of vibrational bands, which complicates precise molecular assignment. This study presents a comprehensive “IR map of the cell” that provides a standardized framework for label-free chemical identification of subcellular compartments across various human cell lines. By integrating Fourier transform infrared (FTIR) spectroscopy with submicron-resolution optical photothermal infrared (OPTIR) microscopy (∼0.3 μm), the cellular landscape was mapped with improved spatial specificity beyond that achievable by conventional FTIR imaging. Advanced chemometric tools were employed to segment the nucleus, cytoplasm, and regions rich in lipids and glycogen, each characterized by a definitive “IR barcode”. Furthermore, *in silico* modeling validated spectral assignments by simulating cellular fingerprints from reference biocompounds, while detailed spectroscopic characterization of subcellular compartments defined marker bands for proteins, lipids, carbohydrates, and nucleic acids. The modeling supported the interpretation that experimental spectra can be approximated as a linear combination of biomolecular classes, helping to constrain spectral band overlap and refine the proposed “IR barcode” for cellular identification. The developed IR map provides a robust, standardized foundation for the label-free interpretation of cellular chemistry. This study demonstrates the utility of IR microscopy as a powerful diagnostic and analytical tool for monitoring metabolic shifts and cellular status at the micrometric level.

## Graphical abstract

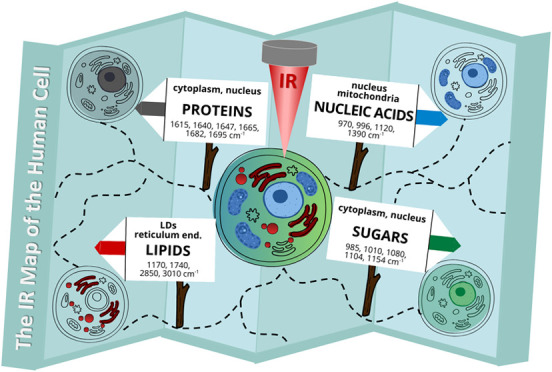

## Introduction

Understanding the cellular structure, function, and molecular composition requires imaging approaches capable of resolving chemically heterogeneous systems. However, the small size, structural complexity, and cell sensitivity to labeling and environmental conditions make this challenging. This underscores the necessity of minimally invasive, chemically specific methods for cellular imaging.

Currently, various modalities are utilized to address these needs. Optical microscopy, including fluorescence and confocal techniques, provides high spatial resolution but relies on a limited number of exogenous labels that may perturb the cellular physiology. Also, fluorescence imaging requires prior selection of a specific probe, involves separate dyes and labeling strategies for each target, and reports only the labeled structures. Electron microscopy provides the nanometer-scale ultrastructure but is typically limited to fixed, dehydrated samples. Mass spectrometry imaging (MSI) offers sensitive, spatially resolved molecular maps but is inherently destructive. In contrast, Raman microscopy enables label-free nondestructive chemical imaging, although signal intensities are governed by the relatively low efficiency of Raman scattering. Consequently, infrared (IR) microscopy serves as a critical, complementary, and nondestructive approach for probing the molecular composition of cells without the need for external markers.

Fourier transform infrared (FTIR) spectroscopy is widely used to correlate biochemical composition with spatial distribution in biological samples. Operating in the mid-infrared (MIR) region, it provides characteristic vibrational signatures of major cellular components and, coupled with IR microscopy, enables microscale chemical imaging in transmission or reflection modes, supporting single-cell analysis.

Numerous studies have demonstrated the utility of IR imaging across diverse biological contexts.^1–3^ Within this context, several spectral band libraries and assignment schemes have been developed to support the interpretation of IR data^4^ typically focusing on major biomolecular classes; however, their direct integration into a unified framework for spatially resolved, subcellular analysis remains limited. Spectral markers related to the protein secondary structure, lipids, and nucleic acids have enabled the discrimination of neuronal and glial cells in neurodegenerative models,^5^ the differentiation of malignant and healthy breast epithelial cells,^6^ the detection of lipid accumulation and oxidative stress in adipocytes,^7^ and the identification of biochemical remodeling during stem cell differentiation^8^ or hypoxia-induced metabolic adaptations.^9^ Such applications highlight the potential of IR imaging for both biomedical diagnostics and mechanistic investigations.

To fully exploit this potential, FTIR cell analysis is frequently performed via focal plane array (FPA)-based hyperspectral imaging. This enables rapid acquisition of thousands of spectra, allowing visualization of biochemical distributions across entire cells. However, spatial resolution remains diffraction-limited (2–10 μm for Cassegrain objectives with NA ≤ 0.65), which is sufficient for resolving major compartments such as the nucleus but inadequate for finer subcellular structures.

To address these limitations, quantum cascade laser (QCL)-based IR microscopy has emerged as a powerful alternative, offering high output power and rapid tuning. This enables targeted measurements up to 3 orders of magnitude faster than conventional broadband FTIR,^10^ although hyperspectral imaging with QCLs can still be time-consuming when scanning multiple discrete frequencies.^11^ QCLs are primarily utilized in optical photothermal infrared (OPTIR) microscopy, where molecular vibrations are induced and detected via a photothermal effect measured by a visible probe. By decoupling spatial resolution from IR diffraction limits, OPTIR achieves submicron (0.3 μm) resolution suitable for probing subcellular chemical heterogeneity. The technique enables imaging of both dried and live cells in aqueous environments, representing a significant advancement for the field.^12,13^ The comparison of FTIR and O-PTIR imaging approaches is depicted in Figure S1 in the Supporting Information (SI).

Advances in single-cell and spatially resolved IR spectroscopy, including synchrotron-based FTIR imaging and OPTIR, have significantly improved spatial resolution and acquisition speed, enabling detailed chemical imaging at the cellular level.^12,14–17^ Despite this progress, a unified framework linking intracellular spectral variability to a comprehensive biomolecular reference system is still lacking. The concept of an “IR map of the cell” introduced in this work aims to address this gap by combining multivariate segmentation with reference-based spectral interpretation, thereby enabling consistent assignment of subcellular regions across different cell types. This highlights the need for approaches that enable subcellular-level resolution while integrating high-resolution imaging with consistent spectral interpretation strategies for single-cell analysis.

In this study, the capabilities of FTIR and OPTIR spectroscopy for single-cell analysis are demonstrated. Their performance is explored with a focus on distinguishing the chemical composition of subcellular compartments. To ensure comprehensiveness, an IR spectroscopic analysis of various human cell lines, including human endothelial cells (HAEC, HBEC), urinary bladder tumor cells (RT4), human brain vascular pericyte (HBVP), and astrocyte (HA), is presented. In contrast to previous studies primarily focused on selected cellular models or descriptive visualization of biochemical heterogeneity, here, we propose a more systematic framework for subcellular IR interpretation across multiple human cell lines. Specifically, a biochemical “IR map of the cell” is constructed by assigning vibrational markers to distinct subcellular classes of biomolecules and linking them to chemically resolved cellular domains. These cellular signatures are systematically compared with the reference spectra of representative proteins, lipids, carbohydrates, and nucleic acids, further supported by multivariate chemometric segmentation and high-resolution OPTIR imaging. In this way, the study goes beyond conventional cell imaging by providing a unified reference-based framework for the biochemical assignment of nucleus-, cytoplasm-, lipid-, and glycogen-related regions. This approach complements Raman-based imaging^18^ and aims to provide a more standardized basis for the interpretation of IR images at the single-cell and subcellular levels.

## Materials and Methods

### FTIR Measurements

ATR-FTIR (attenuated total reflectance–Fourier transform infrared spectroscopy) spectra of reference substances (Table S1) were recorded using an Agilent 670-IR FTIR spectrometer equipped with a single-bounce diamond ATR crystal. Spectra were obtained with a spectral resolution of 4 cm^–1^ in the range of 4000–400 cm^–1^ by coadding 32 scans. For liquid samples, 15 μL of the solution was applied to the ATR crystal and allowed to dry at room temperature for approximately 20 min.

Molecular imaging of cells was performed within 72 h after fixation using an Agilent 670-IR spectrometer combined with a 620-IR microscope (Agilent, USA), following the experimental protocol described previously.^9^

### OPTIR Measurements

OPTIR measurements were conducted using an mIRage-LS microscope (Photothermal Spectroscopy Corporation, Santa Barbara, CA) in copropagation mode with a standard detector. A refractive Cassegrain objective (40×) was employed in conjunction with a MIRcat QT IR laser and a 532 nm probe laser. Single-frequency chemical images were acquired with 0.3 μm step size, and hyperspectra were collected over 980–3000 cm^–1^ with a spectral resolution of 2 cm^–1^.

Cell culturing, sample preparations, and data analysis are summarized in the Supporting Information and Methods.

## Results and Discussion

### Broadband-Based IR Imaging Of Cells

For FPA-based IR imaging, the selection of acquisition modes is critical, as spatial resolution directly dictates the level of observable cellular detail (Figure 1A). While the standard-definition (SD) mode facilitates the rapid acquisition of population-averaged spectra from dozens of cells, it inherently obscures subcellular heterogeneity through spatial averaging. In contrast, ultrahigh-definition (UHD) imaging provides the necessary spatial oversampling of biochemical gradients within a limited field of view (Figure 1B). This enhanced resolution not only enables single-cell detection but also facilitates rigorous region-of-interest (ROI) selection, delivering high-quality spectra representative of the nucleus or a specific metabolite or biochemical substance that can be extracted.

**Figure 1:**
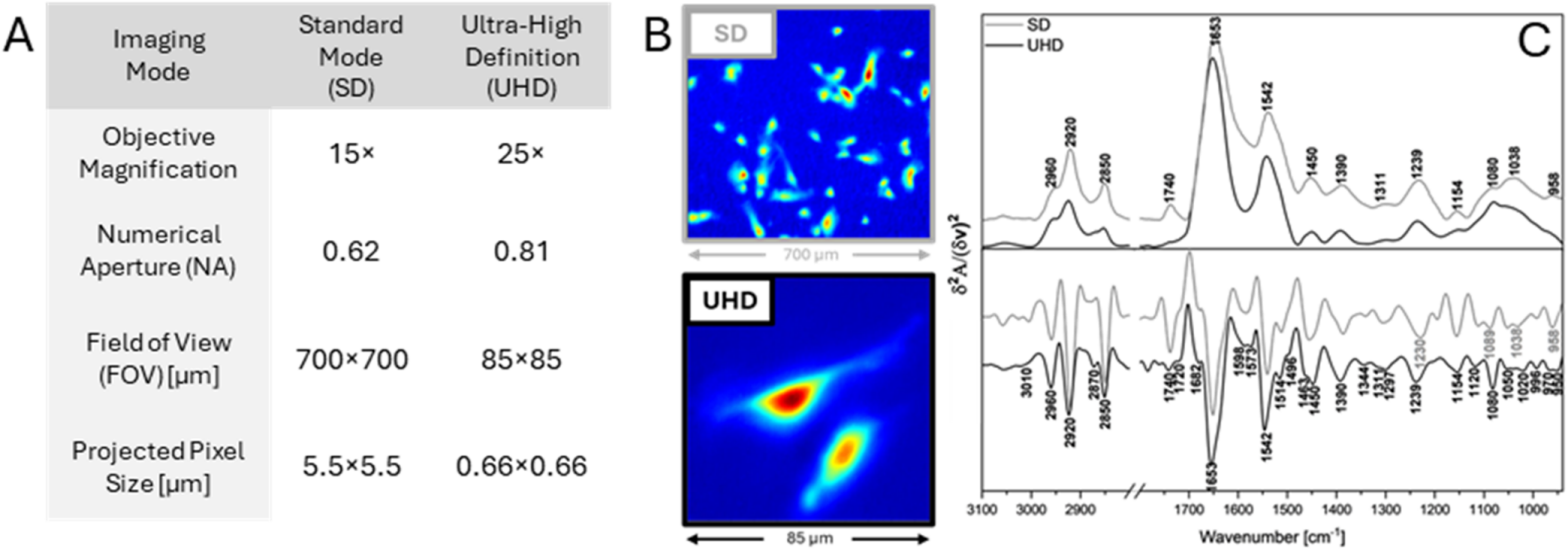
Comparison between SD and UHD imaging modes in FPA-based IR microscopy. (A) Key instrumental parameters. (B) IR images for proteins highlighting differences in spatial resolution. (C) Averaged absorbance spectra and their second derivatives acquired from HBVP cells.

While the overall IR profiles generated from SD and UHD modes (Figure 1C) appear comparable, the UHD mode consistently yields superior spectral clarity, thus facilitating more detailed biochemical analysis of cells. Notably, specific vibrational bands are better resolved under UHD conditions, which is a direct result of minimized spatial blurring. This technical refinement is essential for the detailed biochemical analysis of single cells, ensuring that subtle molecular variations are accurately captured rather than being lost in the population mean.

For the analysis of IR hyperspectral data at the single-cell level, selecting an appropriate chemometric approach is crucial for accurate spatial mapping. Cluster analysis (CA) segments pixels based on spectral similarity to generate spatial chemical maps of subcellular compartments. While often utilized methods, such as hierarchical cluster analysis (HCA) and k-mean cluster analysis (KMCA), provide hard segmentation by assigning each pixel to a single discrete category, these approaches often fail to capture the inherent biochemical complexity of the cell. In contrast, fuzzy cluster analysis (FCA) introduces a “soft partitioning” framework (Figure S2), where a single pixel can belong to multiple clusters. This methodology is especially advantageous for heterogeneous cellular samples, where gradual biochemical transitions between subcellular domains and regions with mixed molecular composition are expected rather than sharp boundaries between cell compartments. By moving away from the rigidity of hard clustering, FCA more accurately reflects the intrinsic molecular overlaps among protein-, lipid-, carbohydrate-, and nucleic acid-rich subcellular regions. This flexibility provides a representation of the subcellular landscape that is better suited for this type of data—gradual biochemical transitions and mixed molecular contributions. The combination of composite images, single FCA components, and corresponding spectra is depicted in Figures S3 and S4 in the SI.

### Subcellular IR Imaging

The application of FCA facilitated the decomposition of the complex cellular hyperspectral cube into chemically distinct domains, enabling the identification of a nuclear region that is spectrally distinct from its surrounding metabolic environment (Figure 2A). The nuclear region was identified by a signature dominated by nucleic acids, while the lipid-rich compartment encompassing the endoplasmic reticulum, membrane-rich structures, and lipid droplets (LDs) was characterized by a pronounced enrichment of high-wavenumber lipid signals. Furthermore, the differentiation of the cytoplasm into two distinct types, that is, thin cellular projections versus organelle-dense regions, highlights the sensitivity of the IR map to subtle shifts in protein and carbohydrate density. Notably, in urothelial RT4 cells, segmentation successfully isolated a glycogen-rich class, underscoring the analytical capability of tracking cell-specific metabolic signatures.

**Figure 2:**
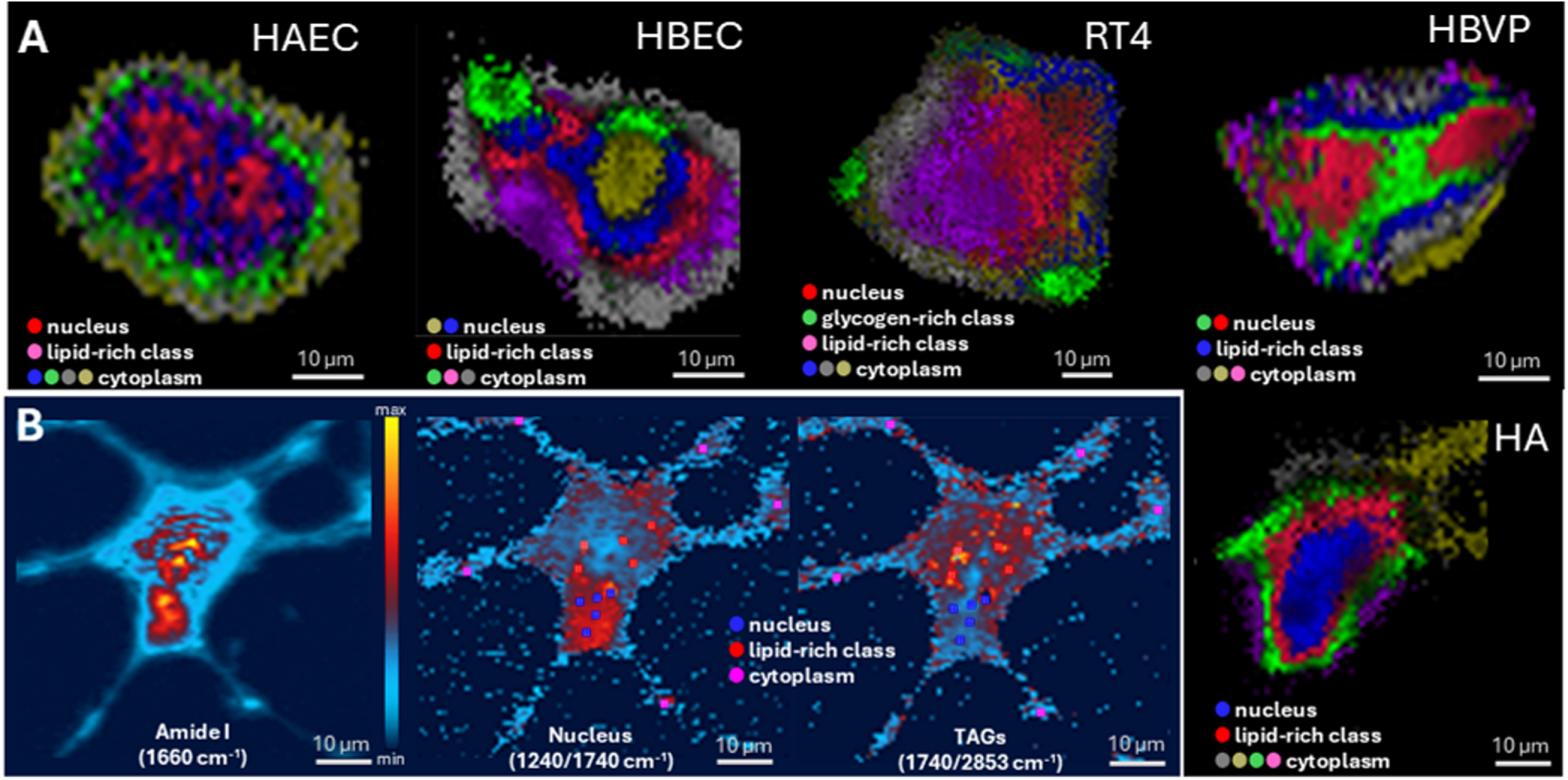
Subcellular segmentation of IR images of cells. (A) False-color FCA maps of UHD IR images showing subcellular compartments (the mean spectra are displayed in Figure S2 in the SI). (B) High-resolution OPTIR imaging of an HA. Left: single-frequency map for proteins (1660 cm^–1^, amide I band). Right: single-frequency ratio maps for the nuclear (1240/1740 cm^–1^, phosphate/lipid contrast) and lipid-rich (1740/2850 cm^–1^, triacylglycerols (TAGs)/total lipid contrast) regions. The full-range OPTIR spectra were acquired from points marked in the ratio maps (Figure S6 in the SI).

A central feature of our approach is the use of nondestructive, label-free IR imaging to identify subcellular components based on their intrinsic spectral signatures. The transition from microscale UHD-FTIR to submicron OPTIR imaging (Figure 2B) provided an important point of comparison for spatial resolution. By utilizing ratio maps at selected frequencies (Figure S5), intracellular domains could be visualized with improved spatial localization compared with classical FTIR microscopy. The specific ratio of 1240/1740 cm^–1^ yielded a sharp contrast for the nuclear region by reducing the influence of local path length variations, while the 1740/2850 cm^–1^ ratio enabled the high-fidelity mapping of triacylglycerols (TAGs) within lipid-rich domains.

To strengthen the interpretative framework, the derived component spectra (Figures S4–S6) were systematically correlated with FTIR spectra of reference biomolecules (Figure 3, Tables S1 and S2). While it is well established that proteins are defined by amide I (1650 cm^–1^) and II (1540 cm^–1^) bands, which correspond to C=O stretching and N–H bending vibrations of the peptide bond, respectively (Figure 3E, Table S2), and lipids are typically identified by CH_2_ and CH_3_ stretching vibrations (2850–2920 cm^–1^) and the ester carbonyl vibration (1735–1740 cm^–1^) (Figure 3D, Table S2); the “IR Map” reveals the nuanced complexity of their spatial overlap. For instance, the concurrent contributions of phospholipid headgroups and DNA/RNA phosphate vibrations at 1230 and 1080 cm^–1^ due to the asymmetric and symmetric PO_2_ stretching, respectively, necessitate the high spatial specificity provided by OPTIR to ensure accurate assignment (Figure 3D,G, Table S2). Carbohydrates and DNA/RNA sugar moieties exhibit the characteristic C–O and C–O–C stretching and bending vibrations in the 1000–1150 cm^–1^ region (Figure 3F, Table S2). Nucleic acids are additionally identified through base-associated ring vibrations appearing between 1500 and 1700 cm^–1^ (Figure 3G, Table S2). By identifying subtle spectral features associated with specific functional groups or structural conformations, this comparative analysis resolves the ambiguities inherent in biological IR spectra, transforming raw vibrational data into a chemically meaningful biochemical map.

**Figure 3:**
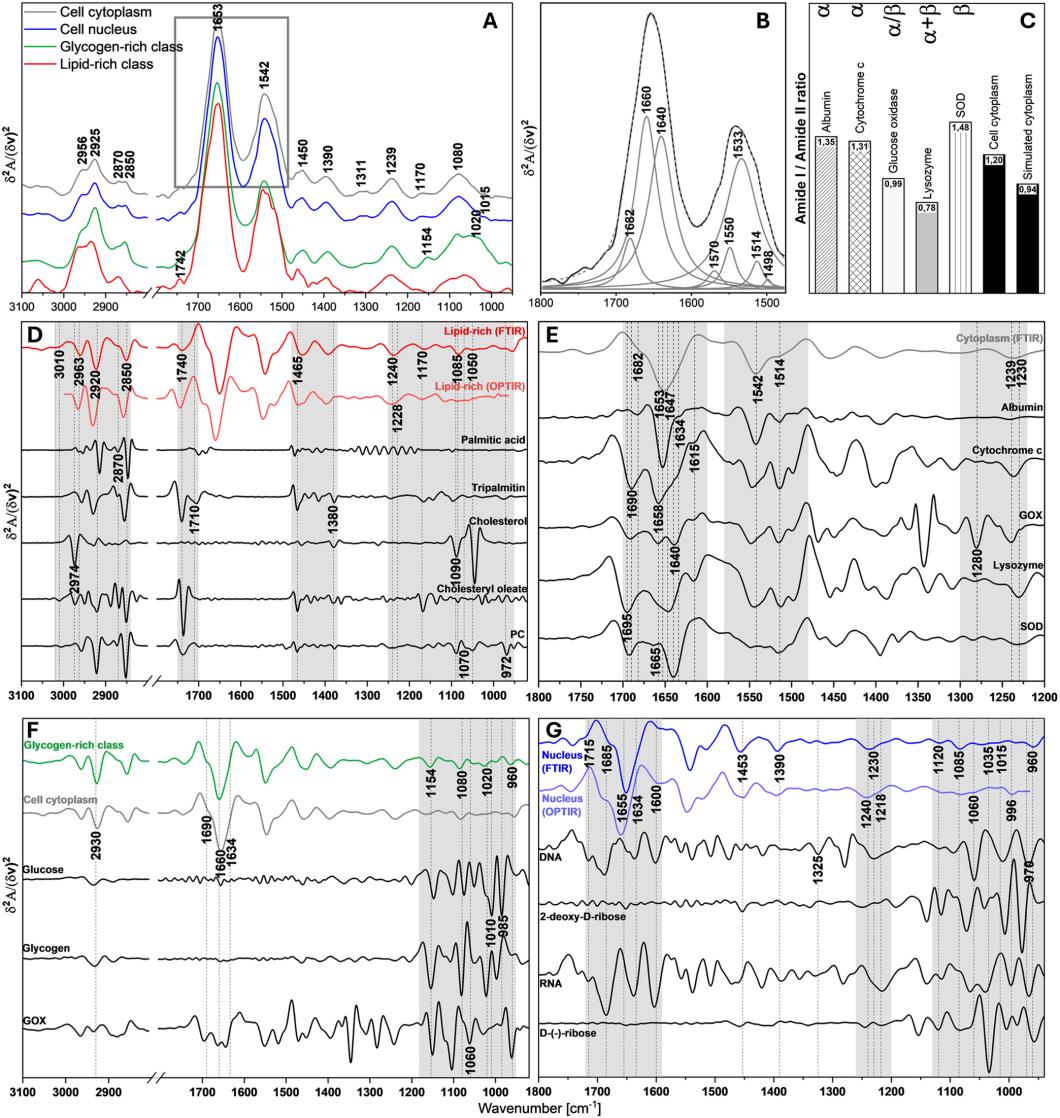
(A) Average second derivative FTIR spectra of the subcellular components from the analyzed cells. (B) Deconvolution of the amide I/II region in the cytoplasm spectrum. (C) Amide I/II ratio was calculated by band integration for cellular, reference, and simulated spectra (see Figure 4). The average FTIR and OPTIR spectra of the HA lipid-rich class (D), cytoplasm (E), glycogen-rich class (F), and nucleus (G) in comparison with the reference spectra. The high-frequency region (3100–2800 cm^–^1) of lipid references was magnified 3× for clarity. The shaded spectral regions indicate the proposed marker region of the biomolecules. The nucleus and cytoplasm spectra are additionally presented, together with the standard deviation; see Figure S7 in the SI.

### Proteins

Proteins, distributed throughout the cell with high concentration in the cytoplasm, are macromolecules composed of amino acids linked by peptide bonds, forming polypeptide chains that adopt characteristic secondary-structure motifs. These conformations, i.e., primarily α-helices, β-sheets, β-turns, and less ordered regions such as loops or random coils, are stabilized by hydrogen bonding and can be rigorously probed via IR spectroscopy.

The amide I band (1600–1700 cm^–1^) arising from C=O stretching serves as the primary indicator of a secondary structure, while amide II (1480–1580 cm^–1^) and amide III (1220–1300 cm^–1^) bands, associated with N–H bending and C–N stretching, respectively, provide complementary structural information.

To establish a reference framework for the “IR map,” proteins with well-characterized signatures were selected according to SCOP/CATH conventions: glucose oxidase (GOX) (α/β), lysozyme (α+β), cytochrome c and albumin (α-rich), and superoxide dismutase (SOD) (β-rich). Proteins are classified by secondary structure content as α-helical-rich, β-sheet-rich, α+β (separate α- and β-regions), or α/β (alternating α-helix−β-strand architectures within a domain). To represent these structural motifs, we selected proteins with well-characterized IR signatures and distinct secondary structures regardless of their biological localization

As shown in Figure 3B, the protein secondary structure is directly reflected in the amide I band shape and position (Figure 3B), with the amide I/amide II intensity ratio serving as a qualitative indicator of the structural order (Figure 3C) as summarized for the reference proteins (Figure 3E).^19^

Albumin, predominantly composed of α-helical structures (1653 cm^–1^), exhibits minimal β-sheet content (1682 cm^–1^). Although not an intracellular protein, its structural uniformity makes it a convenient reference for α-helical signatures. (Figure 3E).^20,21^ A shoulder at 1632 cm^–1^ corresponds to the intramolecular β-sheet structure.^20,21^ The amide I/amide II ratio for α-helical albumin is above 1 (Figure 3C); a similarly high ratio (1.3) was observed for cytochrome c (Figure 3C), whose structure is likewise dominated by α-helices (1658 cm^–1^),^20,21^ with smaller contributions from antiparallel β-sheets (1690, 1640 cm^–1^) and β-turns (1682 cm^–1^) (c.f. Figure 3E).^20,21^ Additional spectral features of the cytochrome c include the amide II band (1542 cm^–1^), ring vibrations of Tyr residue and its phosphorylated derivative (1515 cm^–1^, Figure S8), and amide III vibrations (1239 cm^–1^).^20^

For GOX, an example of α/β proteins, the calculated amide I/II ratio was almost 1 (Figure 3C). The mixed secondary structures are featured by bands corresponding to antiparallel β-sheets (1690, 1640 cm^–1^), α-helices (1658 cm^–1^), and β-turns (1682 cm^–1^, Figure 3E).^20,21^ Lysozyme, representing the α+β class, exhibited the lowest amide I/amide II ratio among the analyzed proteins (Figure 3C). While it contains α-helices, the low ratio implies a significant contribution from random coil structures (1647 cm^–1^). In addition, the presence of both ordered and flexible structures is indicated by amide I bands of antiparallel β-sheets (1695 and 1615 cm^–1^) and α-helices (1653 cm^–1^)^20,21^ (Figure 3E).

Proteins dominated by the β-sheet structure, such as SOD, typically exhibit FTIR absorption peaks at 1690 and 1640 cm^–1^.^20,21^ The SOD spectrum also reveals β-turns at 1665 cm^–1^ (Figure 3E).^20^ The observed high amide I/amide II ratio is attributed to reduced amide II intensity resulting from interactions with metal centers and/or the highly ordered, tightly packed structure, which alter the dipole moment of the peptide bond and affect N–H bond vibrations (Figure 3C).^22^ The FTIR spectrum of the cytoplasm primarily exhibits a protein spectral profile (Figure 3E). Additional bands are observed at ca. 1515 cm^–1^ (Tyr) and in the 1300–1220 cm^–1^ region (amide III vibration). Spectral deconvolution reveals contributions from α-helices (1660 cm^–1^), β-sheets (1640 cm^–1^), and β-turns (1682 cm^–1^) (Figure 3B). This finding is further supported by the amide I/amide II band ratio of 1.2 (Figure 3C), indicating the mixed secondary structures of proteins consistent with the biochemical complexity of the intracellular environment.

As demonstrated above, IR spectroscopy enables the detection of cellular proteins and the characterization of their secondary structures, allowing the analysis of folding, conformation, and intermolecular interactions. All major marker bands and assignments are summarized in Table S2.

### Lipids

Lipids are essential cellular components with diverse structural and functional roles. Key lipidic classes include glycerolipids (TAGs), responsible for the long-term energy storage mainly in the lipid droplets (LDs), sterols (e.g., cholesterol and its storage form, cholesteryl oleate), maintaining membrane stability and supporting cell signaling, and phospholipids that form the cell membranes and maintain their proper fluidity.^23^

The clustering of UHD IR images of the cells distinguishes a lipid-rich class (Figure 2A) recognized by the elevated intensity of the 2750–3050 and 1700–1800 cm^–1^ regions characteristic of lipid moieties (Figure 3D). This class encompasses perilipidic and perinuclear areas and LDs, which are abundant in membranous structures such as the mitochondria and endoplasmic reticulum.^5,7^ OPTIR spectra from these areas exhibit a consistent set of bands (Figure S6). The characteristic IR bands of the major lipid classes observed in the cellular spectra are summarized (Table S2).

Palmitic acid (PA), a primary saturated long chain (C16:0), is a precursor to the biosynthesis of fatty acids (FAs) and a fundamental component of membrane lipids. The most prominent bands in the PA spectrum are assigned to the asymmetric and symmetric stretching vibrations of CH_2_ groups at 2920 and 2850 cm^–1^, respectively (Figure 3D). Stretching vibrations of CH_3_ groups are observed at lower intensity at 2963 and 2870 cm^–1^ (asymmetric and symmetric, respectively). The CH_2_:CH_3_ ratio calculated from the intensities of the 2850 and 2963 cm^–1^ bands serves as an indicator of fatty acyl chain length. The lower the ratio, the shorter the FAs, a shift often observed during lipid peroxidation in stressed cells.^24,25^ Conversely, the sum of CH_2_ and CH_3_ intensities provides an estimate of the total lipid content.^7,8^ Vibrations of these groups are further represented by the 1465 cm^–1^ band arising from in-plane deformations. While the FA carbonyl group exhibits a C=O stretch at 1710 cm^–1^, its esterified counterpart, tripalmitin, shows a red shift to 1740 cm^–1^ (Figure 3D), which is considered a reliable TAG marker in the cellular environment. Deprotonated FAs (COO^–^) are identified by the asymmetric stretching vibration at 1560 cm^–1^, c.f. sodium oleate in Figure S9.

Cholesterol, a paramount component of the cellular lipid bilayer, is represented by three major bands at 2974, 1090, and 1050 cm^–1^ (CH stretching, in-plane bending, and ring deformation modes, respectively)^26^ (Figure 3D). Its storage and transport forms, cholesteryl esters (CEs), are identified by a unique band at 1170 cm^–1^ assigned to asymmetric CO–O–C vibrations.^5^ The spectrum of cholesteryl oleate also exhibits a band at 3010 cm^–1^ (stretching of =CH groups), serving as a marker for unsaturated FA acyl chains.^27^ Among phospholipids, phosphatidylcholine (PC) and phosphatidylethanolamine (PE) are the most abundant in the cellular membranes. PC, primarily located in the outer leaflet, contributes to stability, while PE, found in the inner layer, influences the membrane curvature. These lipids exhibit unique IR features assigned to the asymmetric and symmetric vibrations of the PO_2_ groups. In PC, these bands appear at 1240 and 1080 cm^–1^, whereas in PE, they are downshifted to 1228 and 1070 cm^–1^ (Figures 3D and S9). Additionally, PC can be identified by a unique band at 972 cm^–1^, assigned to the choline group vibration.^28^

By utilizing multiple sets of lipid IR signals, it is possible to identify and quantify key lipid classes within organelles and membranes, while assessing their structure, saturation, and polarity. The IR spectra of the lipid-rich class demonstrate the presence of unsaturated fatty acid chains, FAs, TAGs, phospholipids, and cholesteryl esters (Figure 3D). Several metrics based on these bands’ intensities have been established to evaluate lipid peroxidation in isolated membranes, LD composition changes during differentiation, and the mapping of lipid metabolites, with IR outcomes validated against LC/MS lipidomics or fluorescence staining.^29^

### Carbohydrates

Carbohydrates in mammalian cells function as vital energy sources and structural components, occurring as free glucose, glycogen reserves, and carbohydrate moieties in glycoproteins and glycolipids. Their characteristic IR absorptions are localized in the 900–1200 cm^–1^ region, primarily arising from C–C–O and C–OH stretching and C–OH bending vibrations (Figure 3F),^30^ although these often overlap with phosphate and ester bands. Glucose fuels central metabolic pathways such as glycolysis,^31^ while glycogen forms strongly absorbing, metabolically responsive cytoplasmic regions indicative of cellular activity.^32^ Glycoproteins, essential for cellular signaling, represent complex macromolecules where carbohydrate moieties are covalently linked to protein backbones. To represent these structures, GOX was utilized as a model reference, as its well-characterized FTIR spectrum provides a benchmark for glycoprotein-associated bands.

The primary IR bands of glucose appear at 985 (anomeric C–H, C–CH, and C–CO deformations) and 1010 cm^–1^ (C–C/C–O stretching and in-plane C–OH bending modes). In the cytoplasmic environment, these features are shifted to 960 and 1020 cm^–1^, likely due to structural modifications and molecular interactions within the cellular matrix (Figure 3F). Indicative markers of glycogen are observed at 1020 (C–OH stretching mode), 1080 (C–C stretching and C–OH bending), and 1154 cm^–1^, the latter being assigned to the glycosidic C–O–C stretching and asymmetric ring breathing vibrations (Figure 3F). Although the 1020 cm^–1^ band overlaps with the glucose signal, the simultaneous presence of these three bands is diagnostic for glycogen, as observed in the cytoplasmic granules of RT4 urothelial cancer cells.

Glycoproteins are further identified by the markers 960 and 1154 cm^–1^, as demonstrated by the GOX reference spectrum (Figure 3F). This combination of glycans and proteins exhibits additional robust IR bands at 1060 and 1100 cm^–1^, with the 1000–1100 cm^–1^ region recognized as diagnostic for glycoprotein identification.^33^ The GOX spectrum shows protein amide bands at 1634 cm^–1^ (β-sheets) and 1660 cm^–1^ (α-helices), along with a high-frequency shoulder at 1690 cm^–1^ (β-turns). Cytoplasmic glycosylation is evidenced by IR markers at 960, 1060, and 1154 cm^–1^, where sugar contributions overlap with the protein backbone signature. While CH stretching modes in the 2920–2940 cm^–1^ region are present in all carbohydrates, they cannot serve as a standalone diagnostic feature due to heavy overlap with lipid CH_2_/CH_3_ vibrations.

As demonstrated, IR spectroscopy enables the precise identification and characterization of cellular carbohydrates, demonstrating the utility of vibrational imaging for mapping metabolic processes and the sugar content at the single-cell level. The markers proposed here have been successfully applied to monitor glucose-related metabolism via FTIR and OPTIR imaging in living cells.^16,34^

### Nucleic Acids

Nucleic acids are distributed throughout the cell, encompassing nuclear DNA packaged in chromatin, RNA-rich nucleoli, mitochondrial DNA, and various cytoplasmic RNAs involved in protein synthesis. The FTIR and OPTIR spectra from the nuclear area of the cells are shown in Figure 3G, where they are compared with reference standards: Herring sperm DNA, Torula yeast RNA, deoxy-2-d-ribose, and d-(−)-ribose. Three informative IR regions for nucleic acids lie between 1800 and 800 cm^–1^, with each building block contributing specific vibrational absorption, and are summarized in Table S2.

Nitrogenous bases (purine and pyrimidine) exhibit C=O stretching, skeletal stretching, and NH bending vibrations in the 1600–1800 cm^–1^ region, and their positions of these bands are highly sensitive to base-pairing interactions and hydrogen bonding. The 1600 and 1453 cm^–1^ bands are typically more pronounced in RNA than in DNA. This divergence arises from the presence of the 2′–OH group in ribose, the incorporation of uracil, and the greater conformational flexibility of RNA, all of which enhance the C=N and N–H stretching as well as NH_2_ bending vibrations.

The sugar–phosphate backbone vibrations in the 1250–1000 cm^–1^ region provide critical insights into backbone conformations (Figure 3G). A prominent band observed at 1220–1240 cm^–1^ is attributed to the asymmetric stretching vibration of phosphate groups [ν_as_(PO_2_)]. In RNA, this band shifts to higher wavenumbers due to the distinct phosphate environment and backbone conformation. In DNA, the position of this band is highly sensitive to its structural state (A, B, or Z) and hydration state. While a band at 1218 cm^–1^ is associated with the C–H bending vibration of uracil, it frequently overlaps with the phosphate signal.^35,36^ Below 1100 cm^–1^, the spectrum is predominantly shaped by stretching vibrations of the ribose or deoxyribose rings and the symmetric stretching vibrations of approximately 1085 cm^–1^. Specifically, the phosphate backbone yields markers near 1218 and 1085 cm^–1^ for RNA and 1230 and 1089 cm^–1^ for DNA.

Despite their close structural resemblance, D-ribose and 2-deoxy-d-ribose exhibit distinct spectral signatures owing to the lack of the hydroxyl group in the latter (Figure 3G). For D-ribose, prominent bands appear at 1035 (C–O stretching) and 950 cm^–1^ (ring-related stretching and deformation), both of which are sensitive to sugar puckering.^35^ The corresponding modes for 2-deoxy-d-ribose are observed at 1115, 1070, 1015, and 970 cm^–1^, with the latter serving as a marker of DNA B-, A-, and Z-forms.^35^ Variations of up to 10 cm^–1^ between RNA and DNA are observed (Figure 3G) in the lower-frequency “sugar region” where the discriminating bands are located at 966/996/1035/1070 for RNA and 970/1015/1060 cm^–1^ for DNA.

The FTIR and OPTIR spectra of the cell nucleus primarily reflect a biochemical composition dominated by nucleic acids and nuclear proteins (Figure 3G). However, the 1720–1600 cm^–1^ region is heavily obscured by the amide I and II bands of nuclear proteins. Additional contributions from RNA bases and deprotonated carboxylate groups (likely from proteins or minor lipid residues) are indicated by 1390 and 1218 cm^–1^. Phosphate group vibrations are evident at 1240 and 1085 cm^–1^, while sugar-associated vibrations from ribose and deoxyribose rings appear at 1035, 1015, and 960 cm^–1^. Notably, the distinct RNA marker at 996 cm^–1^ is resolved exclusively in the OPTIR spectra, highlighting the technique’s superior sensitivity. These marker bands have been shown to estimate conformational changes in nuclear DNA and cellular RNA in various contexts, including hypoxia-exposed endothelial cells, hydrated red blood cells, and cancer models.^9,36,37^ Although ribose and 2-deoxyribose provide characteristic vibrational signatures, many of these bands overlap with phosphate and protein modes in cellular spectra. While these nucleic acid-associated pentoses provide characteristic signatures, their utility in cellular spectra is highest when supported by a suite of nucleic acid-specific bands to resolve overlaps with phosphate and protein modes.

### *In Silico* Modeling of Cellular Spectra

To further validate spectral interpretations and assess the contribution of specific biomolecules to the complex IR signals in cells, we simulated the FTIR spectra of the nucleus and cytoplasm. Model spectra were generated by combining normalized second derivative spectra of selected reference compounds in proportions reflecting their expected abundance in cell compartments (details in the SI). This approach addresses the inherent convolution of overlapping vibrational modes in biological spectra and complements unsupervised multivariate techniques that segment biochemically distinct regions without explicitly identifying molecular contributors to specific spectral bands. By simulating the FTIR profiles of key cellular compartments, this study aims to bridge the gap between statistical clustering and molecular assignment, highlighting the dominant molecular classes within the specific spectral regions. The resulting simulations accurately reproduce the primary biochemical signatures observed experimentally (Figure 4 and Table S2).

**Figure 4:**
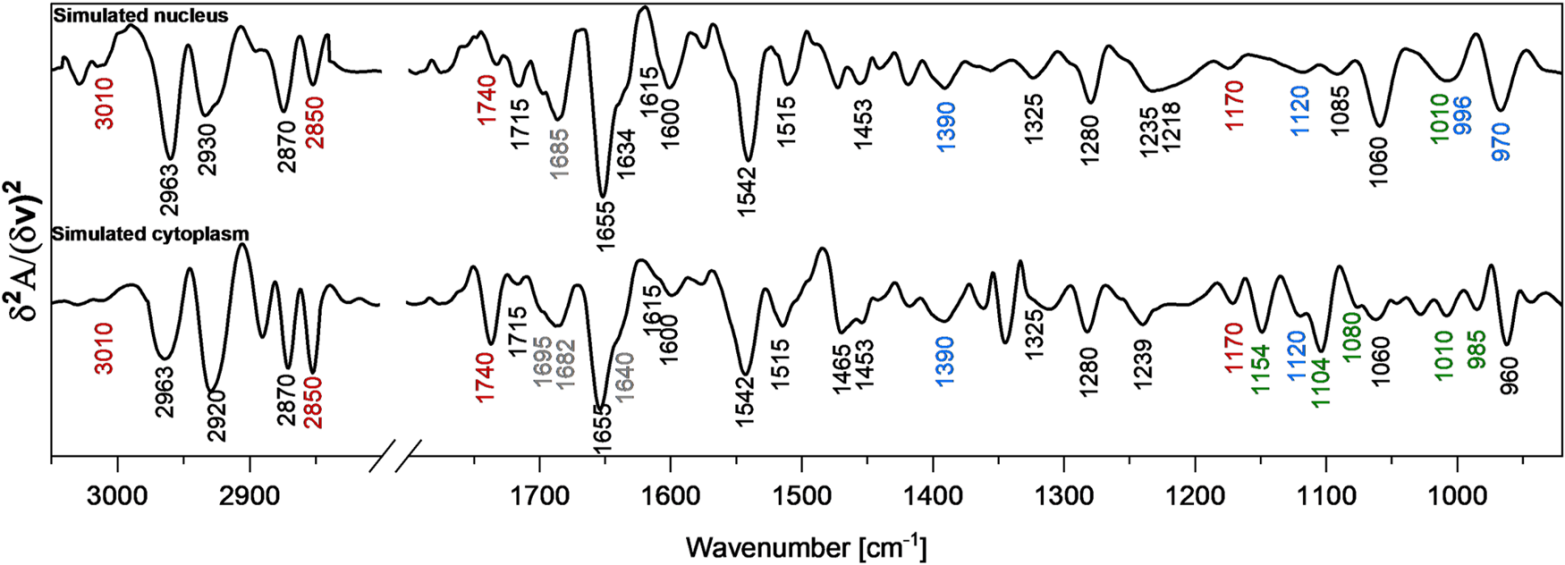
Simulated second derivative IR spectra of the nucleus and cytoplasm generated by a linear combination of reference spectra representing the predominant biomolecular components of each compartment. The color of band positions indicates the biomolecule classes: carbohydrates (green), nucleic acids (blue), lipids (red), and proteins (gray).

The simulated nuclear spectrum reproduced the major nucleic acid- and protein-related bands observed in the experimental data, confirming that a linear combination of DNA, RNA, and protein references can account for the nucleus’s dominant spectral features. Rather than focusing on subtle wavenumber shifts or minor components specific to the model mixture, the simulation was used primarily to distinguish bands influenced by mixed contributions (e.g., overlapping DNA/RNA bases and amide I/II) from those that retain a selective nucleic acid character. Minor discrepancies between the simulated and experimental spectra are expected, as pure reference standards do not capture the full extent of intermolecular interactions, hydration, conformational heterogeneity, or diverse microenvironments present in intact cells. As summarized in Table 1, this comparison supports the assignment of a definitive set of nuclear marker bands that remain diagnostic, despite the presence of complex cellular constituents.

**Table 1:**
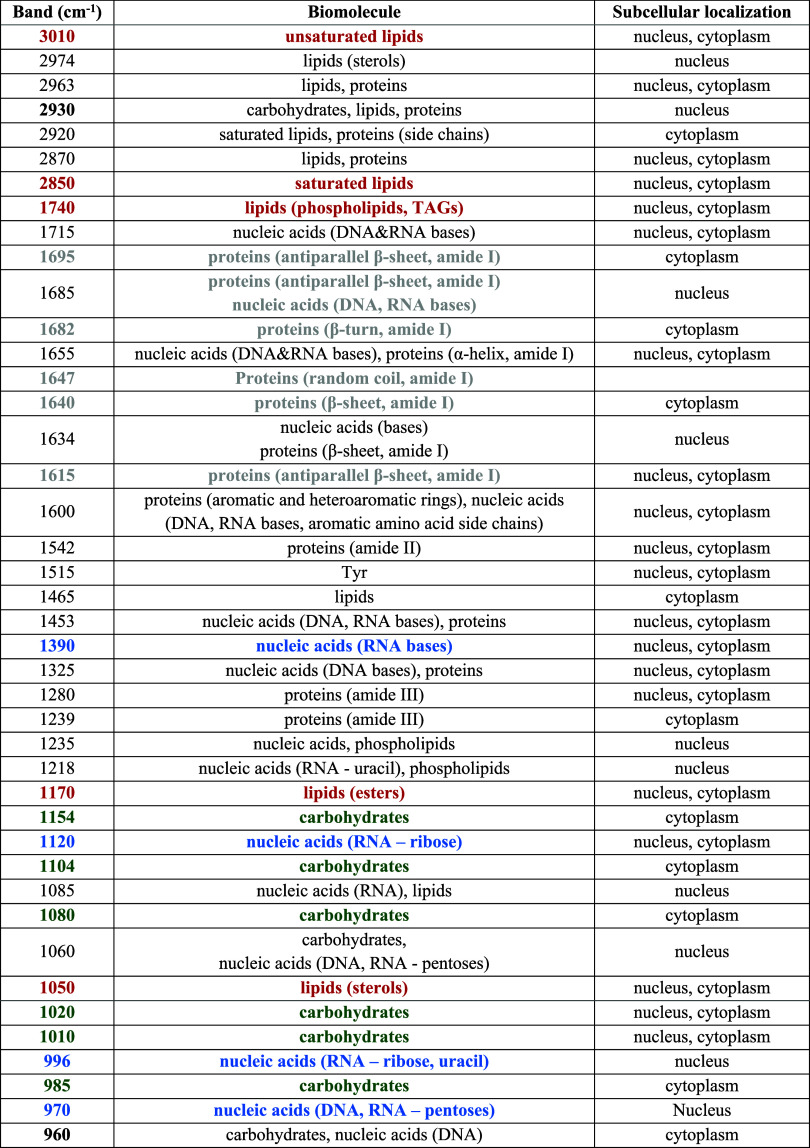
IR Barcode of the Human Cell^a^

a Proposed marker bands for subcellular localization and biochemical assignment. Colors in the manuscript: proteins (gray), lipids (red), carbohydrates (green), and nucleic acids (blue). For vibrations and references, see Table S1.

In the cytoplasmic model, lipid-specific features such as the CH_2_/CH_3_ stretching region and the ester carbonyl band, alongside with the protein amide I/II envelope of proteins, were reproduced with intensities and positions comparable to experimental observation (Figure 4B). The model further reveals a fine structure in the amide and CH stretching regions that are typically smoothed in cellular spectra due to the broad distribution of conformations that the primary experimental bands are well explained by a combination of proteins, lipids, and a minor carbohydrate contribution. This modeling effort facilitates the identification of cellular bands dominated by a single molecular class versus those arising from extensive band convolution, providing a robust analytical foundation for the “**IR map of the cell**” (Table 1).

## Conclusions

In this study, we demonstrate the capabilities of the proposed techniques and methodology by establishing a comprehensive “IR map of the human cell”, providing a standardized and transferable framework for label-free chemical identification of subcellular compartments to support in vitro research applications. By integrating Fourier transform infrared (FTIR) spectroscopy with submicrometer-resolution optical photothermal infrared (OPTIR) microscopy, we demonstrate a combined high-resolution imaging strategy for single-cell analysis, enabling the resolution of cellular heterogeneity with high spatial specificity and partially overcoming the traditional diffraction limits of infrared microscopy. OPTIR additionally offers nondestructive, label-free analysis; faster acquisition when targeting selected spectral features, making it a valuable complementary tool to conventional FTIR imaging; and the potential to measure hydrated biological samples. This capability opens new opportunities for studying cells in a more native state as conventional FTIR typically requires dried specimens.

The use of fuzzy cluster analysis (FCA) enabled chemically meaningful segmentation of major cellular regions, including the nucleus, cytoplasm, and regions characterized by high lipid or glycogen accumulation. It was shown that these compartments possess distinct vibrational signatures that serve as indicative spectral markers for cellular composition. Compared to conventional approaches such as HCA and KMCA, FCA provides a soft classification framework that is better suited to handling mixed spectral contributions and gradual biochemical transitions, resulting in more consistent representations of subcellular organization. A detailed workflow of the proposed analysis (see the algorithm in the SI) has also been provided to facilitate its application and reproducibility. Furthermore, the implementation of biomolecule contribution modeling provided additional support for these spectral assignments. By simulating cellular spectra using linear combinations of reference biomolecules (proteins, lipids, carbohydrates, and nucleic acids), the long-standing challenge of spectral band overlap in biological samples was better constrained and the approach can be used for further compositional analysis.

The results confirm that the IR map complements other imaging modalities, such as Raman spectroscopy, and provides a framework for monitoring metabolic changes and biochemical remodeling at the single-cell level. This framework is expected to facilitate future investigations of cellular responses to environmental factors, drug treatments, and pathological transformations.

## Supplementary Material



## Data Availability

Raw measurement data are available here: 10.57903/UJ/PYNLYQ.

## Notes

Technical details of investigations, mean spectra, band assignments, and spectral analyses (PDF)
