# Supplementary material for: IR Map of the Human Cell
Source: Anal Chem. 2026 Jun 22;98(26):19545–55. doi: 10.1021/acs.analchem.6c00513 (PMC13347700; doi:10.1021/acs.analchem.6c00513)
Supplement: Supplementary file 1 [file ac6c00513_si_001.pdf]

Supporting Information

The IR Map of the Human Cell

Anna Antolak[1], Aleksandra Pragnaca[1,2], Karolina Augustyniak[1], Syeda Takmeel Zahra[1,2], Łukasz Pięta[1], Adrianna Wislocka-Orlowska[1], Katarzyna Majzner[1]*, Malgorzata Baranska[1]*, Kamilla Malek[1]*

[1]Jagiellonian University in Krakow, Faculty of Chemistry, Gronostajowa 2, 30-387, Krakow, Poland
[2]Jagiellonian University in Krakow, Doctoral School of Exact and Natural Sciences, Lojasiewicza 11, Krakow, Poland

Corresponding Authors: K. M.: kamilla.malek@uj.edu.pl; M. B.: m.baranska@uj.edu.pl; K. B. M.: katarzyna.b.majzner@uj.edu.pl

Table of contents

A list of tables 2
A list of figures 2
A list of abbreviations 2
Supplementary Materials and Methods 3
  Cell culture 3
  Preprocessing and data analysis 3
  In silico modelling of nucleus and cytoplasm spectra 4
  Algorithm: FCA cluster segmentation of hyperspectral FTIR images 5
Supplementary Tables 6
Supplementary Figures 9
References 14

A list of tables

Table S1. *The list of reference compounds used to support the spectral band assignments in IR cellular measurements.*

Table S2*. Characteristic IR bands of cellular biomolecules with corresponding assignments. Bold band positions mark key molecular structure markers.*

A list of figures

Figure S1. *Comparison of FTIR and O-PTIR imaging approaches.*

Figure S2*. Examples of multivariate cluster analyses (FCA, HCA, and KMCA) applied to a UHD IR image of a HA cells.*

Figure S3. *Composite image and separated component images resulting from FCA-based image segmentation of the analyzed cells.*

Figure S4. *The FTIR second derivative spectra (UHD mode) of subcellular classes extracted from 6-component FCA analysis of analyzed cells.*

Figure S5. *High-resolution OPTIR imaging of an HA.*

Figure S6. *The average second derivative O-PTIR spectra acquired via hyperspectral point measurements in distinct subcellular regions of a HA cell.*

Figure S7. *The average FTIR spectra of the nucleus and cytoplasm.*

Figure S8. *Second derivative FTIR spectra of L-tyrosine and O-phospho-L-tyrosine.*

Figure. S9 *Second derivative FTIR spectra of sodium oleate and phosphatidylethanolamine.*

A list of abbreviations

CA – Cluster Analysis
CEs – Cholesteryl Esters
DNA – Deoxyribonucleic Acid
FA – Fatty Acid
FCA - Fuzzy Cluster Analysis
FPA – Focal Plane Array
FTIR – Fourier Transform Infrared
GOX – Glucose Oxidase
KMCA – k-Means Cluster Analysis
HA cells – Human Astrocytes
HAEC cells – Human Aortic Endothelial Cells
HBEC cells – Human Brain Endothelial Cells
HBVP cells – Human Brain Vascular Pericytes
HCA – Hierarchical Cluster Analysis
IR – Infrared
LDs – Lipid Droplets
MIR – Mid-infrared
MSI – Mass Spectrometry Imaging
NA – Numeric Aperture
OPTIR - Optical Photothermal Infrared
PA – Palmitic Acids
PBS – Phosphate Buffered Saline
PC – Phosphatidylcholine
PE – Phosphatidylethanolamine
QCL – Quantum Cascade Laser
RNA – Ribonucleic Acid
ROI – Region Of Interest
RT4 – Human Bladder Carcinoma Cell Line
SCOP/CATH – Structural Classification of Proteins
SD mode - Standard-definition
SOD – Superoxide Dismutase
TAGs – Triacylglycerols
Tyr – Tyrosine
UHD mode - Ultra-High-Definition

Supplementary Materials and Methods

Cell culture

For experiments, Human Aortic Endothelial Cells – HAEC (Lonza, Switzerland), Human Brain Endothelial Cells - HBEC (ATCC, USA), Human Bladder Carcinoma Cell Line – RT4 (ATCC, USA), Human Brain Vascular Pericytes – HBVP (ScienCell, USA), and Human Astrocytes – HA (ScienCell, USA) cells were cultured according to the manufacturers' instructions in dedicated media. Cells were seeded directly onto CaF_2 slides (Crystran Ltd, UK) at approximately 150,000 cells per slide. Incubation for 24h allowed sufficient growth and spread, resulting in appropriate confluence. Cells were cultured under standard conditions at 37 °C in a humidified atmosphere containing 5% CO_2. Following incubation, cells were chemically fixed using 2% glutaraldehyde in phosphate-buffered saline (PBS) and subsequently washed with PBS to remove residual fixative. This fixation protocol has previously been shown to preserve biochemical integrity while exerting minimal influence on the molecular features observed in FTIR spectra. Before spectral measurements, the samples were rinsed with deionized water to eliminate PBS residues and air-dried in a laminar flow hood for 24 hours.

Preprocessing and data analysis

Spectral data sets were analysed using a combination of software packages, including CytoSpec (version 2.00.01), MatLab (R2020a, MathWorks, Natick, Ma, USA), PLS_Toolbox (version 9.2.1, Eigenvector Research, Manson, WA, USA), OPUS (version 7.2.139.1294, Bruker, Billercia, MA, USA), and Origin 9.1 (version 2020b, OriginLab Corporation, Northampton, MA, USA). Preprocessing of reference spectra included baseline correction (concave rubberband), second-derivative calculation (Savitzky–Golay algorithm, 2nd-order polynomial, 17 points of smoothing), and vector normalization.

Pre-processing of FTIR cellular images included PCA-based denoising (15 PCs) and smoothing spectra with a Savitzky-Golay algorithm (15 points). In the SD mode. The mean spectrum of the whole cell was obtained using the "define region of interest" function (ROI). In the UHD mode, fuzzy cluster analysis (FCA) was performed in the 920-1770 and 2800-3070 cm-1 regions. Spectral distance was computed using a fuzzy Euclidean metric between the corresponding fuzzy sets, from which six distinct clusters were subsequently extracted. FCA is a non-hierarchical classification method that assigns each pixel a probability (membership value in [0,1]) of belonging to each class. The output of FCM clustering is a membership function that defines the degree to which a given spectrum belongs to the clusters. In the similarity maps assembled by FCM clustering, the membership values are encoded by the colormap, specifically by color intensity in single-color colormaps. The single-cluster image is plotted on the axes of the preprocessed maps using the colormap, and a composite image can subsequently be generated. FCA visualizes cluster membership continuously, effectively displaying component contributions with gradual intensity variations. Based on the distribution maps of proteins (1620–1680 cm^{-1}), cells' spectra from a single class were averaged and extracted. FTIR spectra were baseline-corrected (10 iterations), transformed into a second derivative (Savitzky–Golay algorithm, 2nd order polynomial, 17 points of smoothing), and normalized (unit vector normalization) in the whole spectral range. The band deconvolution of amide I-II bands in cytoplasmic spectra was performed in Origin 9.1 using the Lorentz function in the range of 1500-1800 cm^{-1} to separate the complex Amide I band into individual components. This analysis enabled determination of the position, peak height, and width of each component, allowing a more precise interpretation of the results.

Single-frequency OPTIR chemical images were acquired at 1240, 1540, 1660, 1740, 2850, and 2960 cm^{-1}. The ratio images for 1240/1740 cm^{-1} and 1740/2850 cm^{-1} were generated from single-frequency chemical images. Additionaly, the ratio images for 2850/2960 cm^{-1} (relative contribution of CH_2 to CH_3 groups and is commonly associated with lipid chain length and packing order), 1660/2850 cm^{-1} (relative contribution of protein-to-lipid content), and 1535/1660 cm^{-1} (Amide II/Amide I ratio) were generated to offer insight into protein composition and structural variations. Based on these ratios, hyperspectra were collected in regions of interest. The OPTIR full hyperspectra covering the 965–3000 cm^{-1} range were then extracted from defined regions of interest, including cytoplasmic extensions. OPTIR spectra were baseline-corrected (10 iterations), transformed into a second derivative (Savitzky–Golay

algorithm, 2nd order polynomial, 17 points of smoothing), and normalized (unit vector normalization) in the whole spectral range

In silico modelling of nucleus and cytoplasm spectra

Spectral modeling was performed in Origin 9.1 using second-derivative spectra to minimize baseline contributions and enhance the resolution of overlapping bands. The proportions of each component were selected based on literature data and biological reasoning, with an emphasis on using representative molecules that best mimic the biochemical and structural properties of their cellular counterparts. The model of the nucleus comprised approximately 35% DNA, predominant nucleic acid component, 15% RNA, 45% proteins modeled by albumin, serving as a proxy for histones, that represents a globular, folded structure, similar to typical nuclear enzymes and transcription factors and 5% lipids in the form of phosphatidylcholine (PC), reflecting the lipid content of the nuclear envelope. The cytoplasm model included 60% proteins represented by glucose oxidase, chosen for its well-defined amide bands and globular conformation characteristic of cytoplasmic enzymes, 15% RNA, accounting for mRNA and ribosomal RNA, 15% lipids modeled by cholesteryl oleate to represent neutral lipids and lipid droplets more relevant to cytoplasmic storage lipids than membrane phospholipids, and 10% sugars as glucose, reflecting free monosaccharides involved in cellular energy metabolism.

Algorithm: FCA cluster segmentation of hyperspectral FTIR images

Input:
- hyperspectral FTIR dataset
- spectral resolution: 4 cm-1

Image preprocessing:

Quality test:
- criterion: integral absorption as a measure for sample thickness
- spectral region: 1620-1680 cm-1
- lower limit - set empirically to exclude spectra with too low absorptions from further multivariate analysis

PCA-based noise reduction:
- number of principal components: 15

Smoothing:
- Savitzky-Golay algorithm
- smoothing points: 15

Cut:
- in spectral dimensions
- region to keep: 920-3100 cm^{-1}

Normalization:
- type: vector normalization
- spectral region: 920-3100 cm^{-1}

Fuzzy C-Means Cluster Imaging:
- number of clusters: 6
- distance method: Euclidean
- stop criterion: 0.0005
- generate composite image

Dominant spectral features:

Nucleus:
- phosphate vibrations (1230,1080 cm^{-1})
- base-associated ring vibrations (1500-1700 cm^{-1})
- amide I (1650 cm^{-1}) & amide II (1540 cm^{-1})

Lipid-rich class:
- CH_2/CH_3 stretching vibrations (2850–2920 cm^{-1})
- ester carbonyl vibration (1740 cm^{-1})

Carbohydrates:
- C–O and C–O–C stretching and bending vibrations (1000–1150 cm^{-1})

Cytoplasm (lower intensity):
- amide I (1650 cm^{-1}) & amide II (1540 cm^{-1})
- CH_2/CH_3 stretching vibrations (2850–2920 cm^{-1})
- carbohydrates

Supplementary Tables

Table S1. *The list of reference compounds used to support the spectral band assignments in IR cellular measurements.*

**Class of Compounds**	**Reference Compounds**	**State of Sample**	**Company**
**Proteins**	Albumin (bovine serum)	Solution	SigmaAldrich
	Cytochrome c (bovine heart)	Solution	SigmaAldrich
	Glucose oxidase	Solid	SigmaAldrich
	LysosymeLysozyme	Solid	SigmaAldrich
	Superoxide dismutase (SOD)	Solid	SigmaAldrich
	L_tyrosine	Solid	SigmaAldrich
	O-phospho-L-tyrosine	Solid	SigmaAldrich
**Glycoproteins**	Glucose oxidase	Solid	SigmaAldrich
**Lipids**	Palmitic acid	Solid	SigmaAldrich
	Tripalmitin	Solid	Supelco
	Cholesterol	Solid	SigmaAldrich
	Cholesteryl oleate	Solid	SigmaAldrich
	L-α-phosphatidylcholine (PC)	Solid	SigmaAldrich
	Sodium oleate	Solid	SigmaAldrich
	L-α-phosphatidylethanolamine (egg yolk)	Solid	SigmaAldrich
**Nucleic Acids**	DNA (herring sperm)	Solution	SigmaAldrich
	RNA (torula yeast)	Solution	SigmaAldrich
	2-deoxy-D-ribose	Solution	SigmaAldrich
	D-(-)-ribose	Solution	SigmaAldrich
**Carbohydrates**	D-(+)-glucose	Solid	SigmaAldrich
	Glycogen (bovine liver)	Solid	SigmaAldrich

Table S2. *Characteristic IR bands of cellular biomolecules with corresponding assignments. Bold band positions mark key molecular structure markers. Abbreviations: δ – deformation; υ – stretching (s – symmetric; as – asymmetric).*

Band (cm^{-1})	Assignment	Biomolecule	Reference
**3010**	ν(=C–H)	**unsaturated lipids**	[1]
2974	ν(CH_3), υ(CH_2)	lipids (sterols)	[2]
2963	$ν_{as}$(CH_3)	lipids, proteins	[3]
2930	ν(CH/CH_2)	carbohydrates, lipids, proteins	[4][5]
2920	$ν_{as}$(CH_2)	saturated lipids, proteins (side chains)	[3]
2870	$ν_s$(CH_3)	lipids, proteins	[3]
**2850**	$ν_s$(CH_2)	**saturated lipids**	[3]
**1740**	ν(C=O)	**lipids (phospholipids, TAGs)**	[6]
1715	ν(C=O)	nucleic acids (DNA&RNA bases)	[7][8][9]
1710	ν(C=O)	lipids	[6]
**1695**	ν(C=O), δ(N-H)	**proteins** (antiparallel β-sheet, amide I)	[10][11][12]
**1690**	ν(C=O), δ(N-H)	**proteins** (antiparallel β-sheet, amide I)	[10][11][12][13]
1690–1680	ν(C=O), δ(N–H)	nucleic acids (DNA, RNA bases)	[7][8]][9][14]
**1682**	ν(C=O), δ(N-H)	**proteins** (β-turn, amide I)	[10][11][12]
**1665**	ν(C=O), δ(N-H)	**proteins** (β-turn, amide I)	[10][11]
1660-1650	ν(C=C), ν(C=O)/δ(N–H)	nucleic acids (DNA&RNA bases), proteins (α-helix, amide I)	[7][8][9][10][14] [12][13]
**1647**	ν(C=O)/δ(N–H)	**proteins** (random coil, amide I)	[10][11]
**1640**	ν(C=O)/δ(N-H)	**proteins** (β-sheet, amide I)	[10][11]
1634	ν(C=C), ν(C=O), δ(N–H)	nucleic acids (bases)<br>proteins (β-sheet, amide I)	[8][9][14]<br>[10][11][12][13]
**1615**	ν(C=O), δ(N-H)	**proteins** (antiparallel β-sheet, amide I)	[11]
1600	ν(C–C) ring, (C=N), ν(N–H), δ(–NH_2)	proteins (aromatic and heteroaromatic rings), nucleic acids (DNA, RNA bases, aromatic amino acid side chains)	[8][14]
1560	ν(-COO^-)	lipids	[15]
1542	δ(N-H), ν(C-N)	proteins (amide II)	[13]
1515	ν(CC)	Tyr	[12]
1465	δ(CH_2) scissoring	lipids	[6]
1453	ν(C–N) bases, ν-ring uracil, δ(CH_3), δ(CH_2)	nucleic acids (DNA, RNA bases), proteins	[9][16][17]
**1390**	δ(C–H)	**nucleic acids (RNA bases)**	[8][9][18]
1380	ν(-COO^-)	lipids	[6]
1325	ν(C–N), δ(N–H)	nucleic acids (DNA bases), overlaps with protein modes	[8][17][18]
1280	ν(C-N), δ(N-H), ν(CH_3-C)	proteins (amide III)	[10][11]
1240-1220	$ν_{as}$(PO_2)	nucleic acids, phospholipids	[7][8][9][14][15][19]
1240-1230	ν(C-N), δ(N-H), ν(CH_3-C)	proteins (amide III)	[10][11]
1218	δ(CH)	nucleic acids (RNA - uracil), phospholipids	[7][16]
**1170**	$ν_{as}$(CO–O–C)	**lipids (esters)**	[1]
**1154**	ν(COC), $ν_{as}$(CC); ring breathing	**carbohydrates**	[4][20]
**1120**	ν(C–O)	**nucleic acids (RNA – ribose)**	[8][9]
1104	ν(CO) ring deformation	carbohydrates	[4][5]
1090	δ(C-H)	lipids	[2]
1085	$ν_s$(PO_2)	nucleic acids (RNA), lipids	[6][7][8][9]
**1080**	β(COH),ν(C-C)	**carbohydrates**	[4][5][20]

Table S2. *Characteristic IR bands of cellular biomolecules with corresponding assignments. Bold band positions mark key molecular structure markers. Abbreviations: δ – deformation; υ – stretching (s – symmetric; as – asymmetric).*

Band (cm^{-1})	Assignment	Biomolecule	Reference
1070–1050	ν(C–O)	nucleic acids (DNA, RNA - pentoses)	[7][8][9][14]
1070	$\nu_s(PO_2)$	lipids	[6]
1060	ν(COH) deformation	carbohydrates	[4][5]
**1050**	(C–C–O) ring deformation	**lipids** (sterols)	[2]
**1035**	ν(C–O)	nucleic acids (DNA, RNA – pentoses)	[7][8]
**1020**	ν(COH)	**carbohydrates**	[4][20]
1015	ν(C–O)	nucleic acids (DNA, RNA – pentoses)	[7][8][4]
**1010**	ν(CC), ν(CO), β(COH)	**carbohydrates**	[4]
**996**	ν(C–C), ν(C–O), ν and δ ring	nucleic acids (RNA – ribose, uracil)	[7][8][9][14][16]
**985**	C–H deformation (β-anomer), β(CCH), β(CCO)	**carbohydrates**	[4]
972	$\nu_{as}(CH_3)_3N^+$	lipids (phospholipids)	[6]
**970**	ν(C–C), ν(C–O)<br>ν sugar-phosphate backbone	**nucleic acids (DNA, RNA – pentoses)**	[7][8][9][14]
960	ν_s(C–O) ν(C–C),β(COH)	carbohydrates, nucleic acids (DNA)	[4][5][8][9]

Supplementary Figures

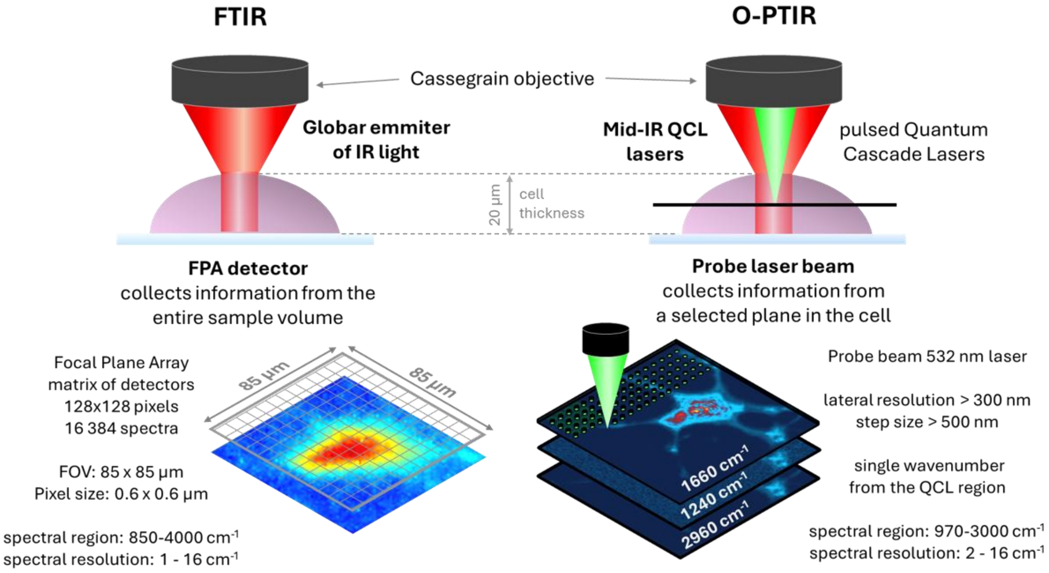

Figure S1. *Comparison of FTIR and O-PTIR imaging approaches. In FTIR, a globar serves as a broadband IR emitter, and the signal is collected using an FPA-based detector, yielding an image acquired over the entire field of view, where each pixel (voxel) contains a spectrum integrated through the sample thickness. In contrast, O-PTIR employs a tunable QCL as the IR excitation source, with detection via a focused probe laser. The measurement is performed at selected single wavenumbers, with the laser probing discrete points within a defined focal plane, enabling spatially localized signal collection.*

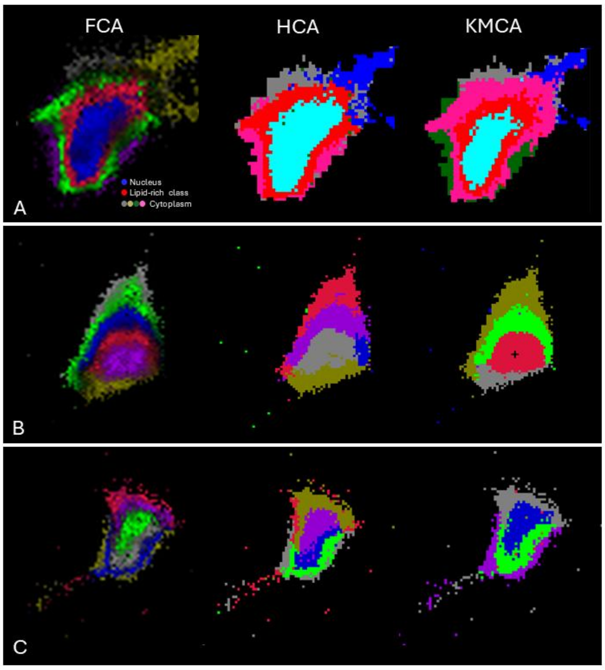

Figure S2. *Examples of multivariate cluster analyses (FCA, HCA, and KMCA) applied to a UHD IR image of HA cells, illustrating the differences between hard and soft clustering approaches. The identified components highlight distinct cellular regions, including the nucleus, lipid-rich domains, and cytoplasm, with soft clustering revealing overlapping memberships between components.*

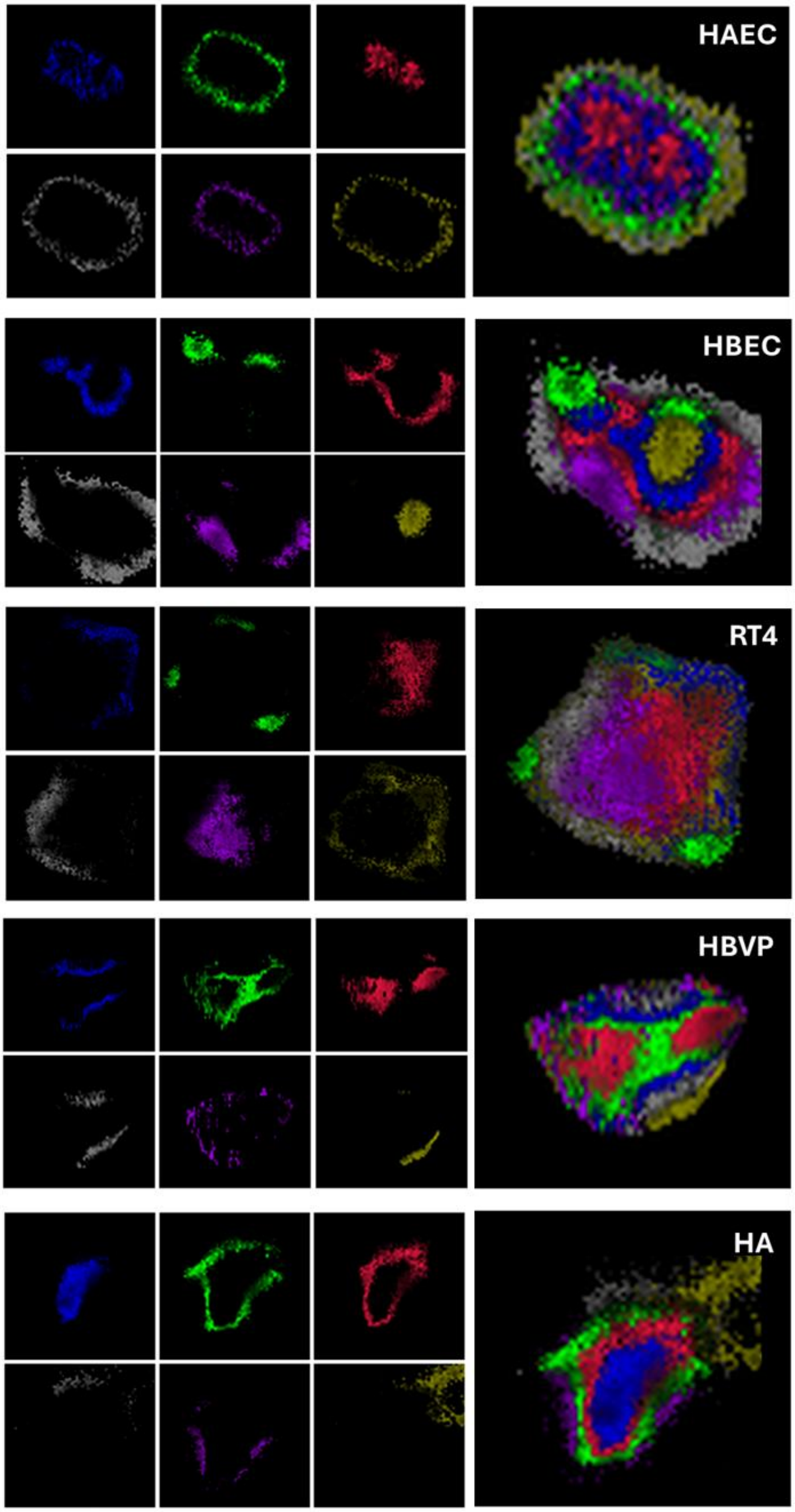

Figure S3. *Composite images and separated component images resulting from FCA-based image segmentation of the analyzed HAEC (**A**), HBEC (**B**), RT4 (**C**), HBVP (**D**), HA (**E**) cells.*

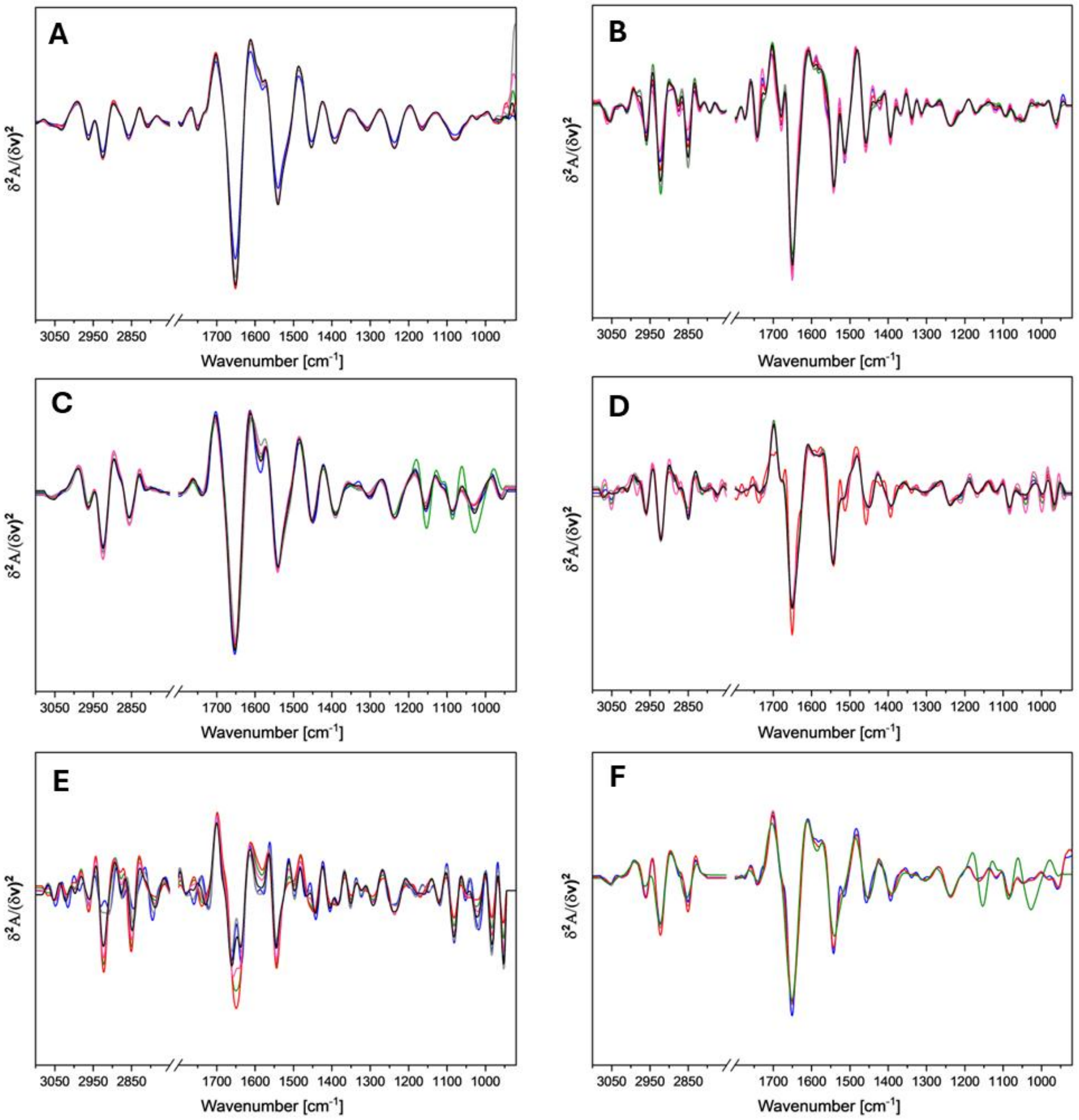

Figure S4. A-E. *The FTIR second derivative spectra (UHD mode) of subcellular classes extracted from 6-component FCA analysis of HAEC (**A**), HBEC (**B**), RT4 (**C**), HBVP (**D**), HA (**E**) cells. Color code corresponds to the colors of FCA components in **Fig. 2.; F**. Average spectra for the subcellular classes obtained from the analyzed cells: cytoplasm (grey), nucleus (blue), lipid-rich class (red), glycogen-rich class (green).*

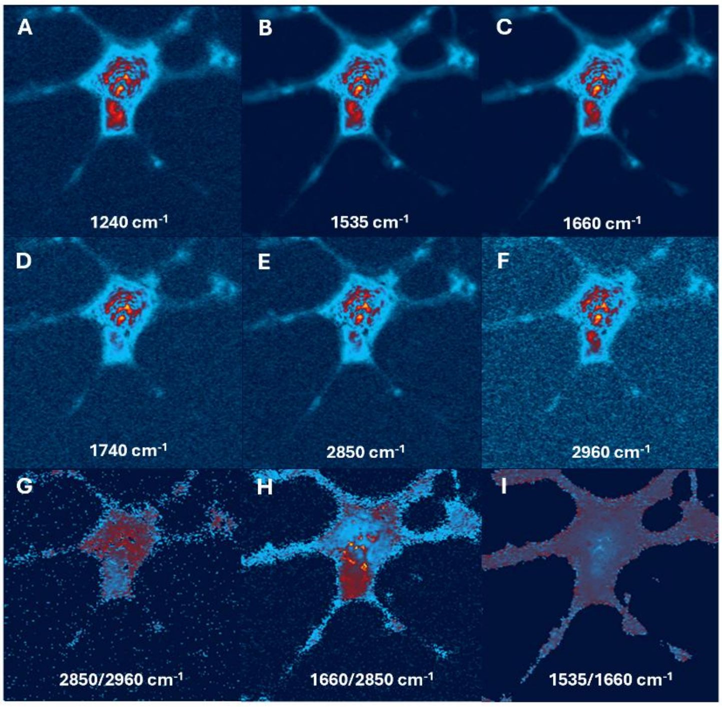

Figure S5. *High-resolution OPTIR imaging of an HA. **A-F**: single-frequency map for 1240, 1535, 1660, 1741, 2850, and 2960 cm-1, respectively. **G-I**: Single-frequency ratio maps for 2850/2960 cm⁻¹, 1660/2850 cm⁻¹, and 1535/1660 cm⁻¹.*

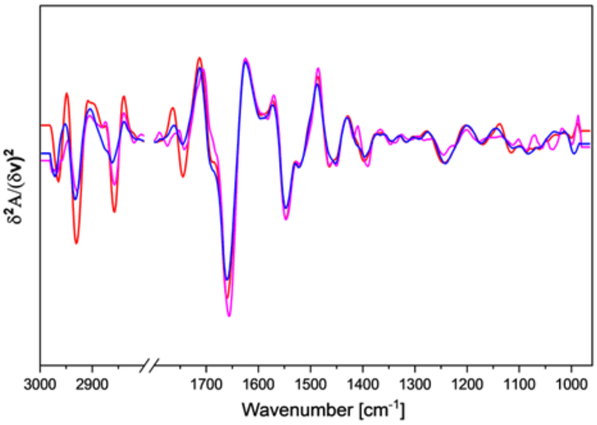

Figure S6. *The average second derivative O-PTIR spectra acquired via hyperspectral point measurements in distinct subcellular regions of a HA cell: nucleus (blue), lipid-rich area (red), and cytoplasmic protrusions (pink). Color code corresponds to the legend in **Fig. 2.B**.*

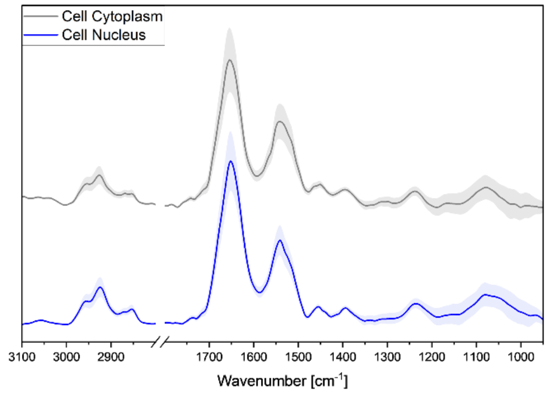

Figure S7. *The average FTIR spectra of the nucleus (blue) and cytoplasm (grey). Spectra averaged for subcellular components from the analyzed cells (HAEC, HBEC, RT4, HBVP, HA cell lines), including standard deviation (SD).*

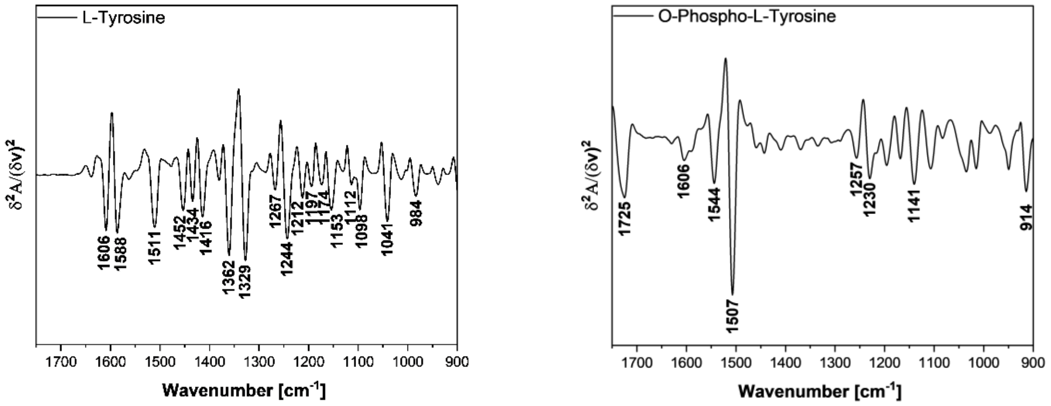

Figure S8 *Second derivative FTIR spectra of L-tyrosine and O-phospho-L-tyrosine. Spectral range: 1750-1200 cm^{-1}. Most L-tyrosine bands overlap with the Amide bands of proteins in the cell spectrum, except the 1511 cm^{-1} band. The phosphorylation of this amino acid residue appears in several dysfunctional properties and can be recognized in the cell spectrum by a 4-6 cm^{-1} down shift of the 1511 cm^{-1} band.*

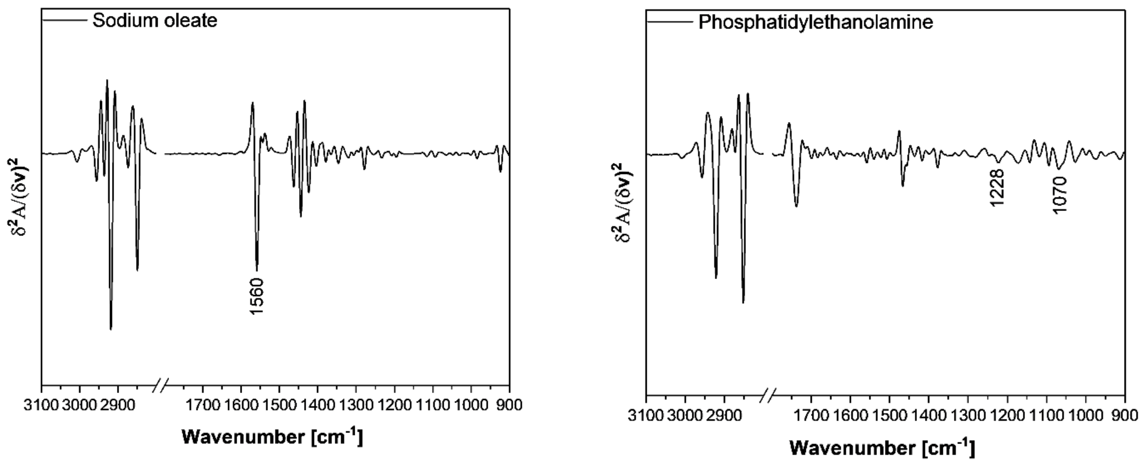

Figure S9 *Second derivative FTIR spectra of sodium oleate and phosphatidylethanolamine. Spectral range: 3100-900 cm^{-1}.*

References

(1) Wrobel, T.; Mateuszuk, L.; Chlopicki, S.; Malek, K.; Baranska, M. Imaging of Lipids in Atherosclerotic Lesion in Aorta from ApoE/LDLR -/- Mice by FT-IR Spectroscopy and Hierarchical Cluster Analysis. *Analyst* **2011**, *136* (24). https://doi.org/10.1039/c1an15311k.

(2) Gupta, U.; Singh, V.; Kumar, V.; Khajuria, Y. Spectroscopic Studies of Cholesterol: Fourier Transform Infra-Red and Vibrational Frequency Analysis. *Mater. Focus* **2014**, *3* (3). https://doi.org/10.1166/mat.2014.1161.

(3) Staniszewska, E.; Malek, K.; Baranska, M. Rapid Approach to Analyze Biochemical Variation in Rat Organs by ATR FTIR Spectroscopy. *Spectrochim. Acta - Part A Mol. Biomol. Spectrosc.* **2014**, *118*. https://doi.org/10.1016/j.saa.2013.09.131.

(4) Wiercigroch, E.; Szafraniec, E.; Czamara, K.; Pacia, M. Z.; Majzner, K.; Kochan, K.; Kaczor, A.; Baranska, M.; Malek, K. Raman and Infrared Spectroscopy of Carbohydrates: A Review. *Spectrochimica Acta - Part A: Molecular and Biomolecular Spectroscopy*. 2017. https://doi.org/10.1016/j.saa.2017.05.045.

(5) Azimzadeh Andarabi, E.; Norouzian-Alam, S.; Shayganmanesh, M.; Haji Abdolvahab, M. Analysis of Glucose Concentrations in Blood Solutions Using FTIR and Raman Spectroscopy Methods. *Biomed. Opt. Express* **2025**, *16* (7). https://doi.org/10.1364/boe.561552.

(6) Faramarzi, B.; Moggio, M.; Diano, N.; Portaccio, M.; Lepore, M. A Brief Review of FT-IR Spectroscopy Studies of Sphingolipids in Human Cells. *Biophysica*. 2023. https://doi.org/10.3390/biophysica3010011.

(7) Benedetti, E.; Bramanti, E.; Papineschi, F.; Rossi, I.; Benedetti, E. Determination of the Relative Amount of Nucleic Acids and Proteins in Leukemic and Normal Lymphocytes by Means of Fourier Transform Infrared Microspectroscopy. *Appl. Spectrosc.* **1997**, *51* (6). https://doi.org/10.1366/0003702971941304.

(8) Dovbeshko, G. I.; Gridina, N. Y.; Kruglova, E. B.; Pashchuk, O. P. FTIR Spectroscopy Studies of Nucleic Acid Damage. In *Talanta*; 2000; Vol. 53. https://doi.org/10.1016/S0039-9140(00)00462-8.

(9) Banyay, M.; Sarkar, M.; Gräslund, A. A Library of IR Bands of Nucleic Acids in Solution. *Biophysical Chemistry*. 2003. https://doi.org/10.1016/S0301-4622(03)00035-8.

(10) Yang, H.; Yang, S.; Kong, J.; Dong, A.; Yu, S. Obtaining Information about Protein Secondary Structures in Aqueous Solution Using Fourier Transform IR Spectroscopy. *Nat. Protoc.* **2015**, *10* (3). https://doi.org/10.1038/nprot.2015.024.

(11) Usoltsev, D.; Sitnikova, V.; Kajava, A.; Uspenskaya, M. Systematic FTIR Spectroscopy Study of the Secondary Structure Changes in Human Serum Albumin under Various Denaturation Conditions. *Biomolecules* **2019**, *9* (8). https://doi.org/10.3390/biom9080359.

(12) Goormaghtigh, E.; Ruysschaert, J. M.; Raussens, V. Evaluation of the Information Content in Infrared Spectra for Protein Secondary Structure Determination. *Biophys. J.* **2006**, *90* (8). https://doi.org/10.1529/biophysj.105.072017.

(13) Goormaghtigh, E.; Gasper, R.; Bénard, A.; Goldsztein, A.; Raussens, V. Protein Secondary Structure Content in Solution, Films and Tissues: Redundancy and Complementarity of the Information Content in Circular Dichroism, Transmission and ATR FTIR Spectra. *Biochim. Biophys. Acta - Proteins Proteomics* **2009**, *1794* (9). https://doi.org/10.1016/j.bbapap.2009.06.007.

(14) Tsuboi, M. Application of Infrared Spectroscopy to Structure Studies of Nucleic Acids. *Appl. Spectrosc. Rev.* **1970**, *3* (1). https://doi.org/10.1080/05704927008081687.

(15) Cai, J.; Deng, J.; Wen, S.; Zhang, Y.; Wu, D.; Luo, H.; Cheng, G. Surface Modification and Flotation Improvement of Ilmenite by Using Sodium Hypochlorite as Oxidant and Activator. *J.*

Mater. Res. Technol. **2020**, *9* (3). https://doi.org/10.1016/j.jmrt.2020.01.031.

(16) Susi, H.; Ard, J. S. Vibrational Spectra of Nucleic Acid Constituents-I. Planar Vibrations of Uracil. *Spectrochim. Acta Part A Mol. Spectrosc.* **1971**, *27* (9). https://doi.org/10.1016/0584-8539(71)80211-8.

(17) Letellier, R.; Ghomi, M.; Taillandier, E. Interpretation of Dna Vibration Modes Ii-the Adenosine and Thymidine Residues Involved in Oligonucleotides and Polynucleotides. *J. Biomol. Struct. Dyn.* **1987**, *4* (4). https://doi.org/10.1080/07391102.1987.10507667.

(18) Liquier, J.; Akhebat, A.; Taillandier, E.; Ceolin, F.; Dinh, T. H.; Igolen, J. Characterization by FTIR Spectroscopy of the Oligoribonucleotide Duplexes r(A-U)6 and r(A-U)8. *Spectrochim. Acta Part A Mol. Spectrosc.* **1991**, *47* (2). https://doi.org/10.1016/0584-8539(91)80089-2.

(19) Zucchiatti, P.; Mitri, E.; Kenig, S.; Bille, F.; Kourousias, G.; Bedolla, D. E.; Vaccari, L. Contribution of Ribonucleic Acid (RNA) to the Fourier Transform Infrared (FTIR) Spectrum of Eukaryotic Cells. *Anal. Chem.* **2016**, *88* (24). https://doi.org/10.1021/acs.analchem.6b02744.

(20) Hackett, M. J.; Sylvain, N. J.; Hou, H.; Caine, S.; Alaverdashvili, M.; Pushie, M. J.; Kelly, M. E. Concurrent Glycogen and Lactate Imaging with FTIR Spectroscopy to Spatially Localize Metabolic Parameters of the Glial Response Following Brain Ischemia. *Anal. Chem.* **2016**, *88* (22). https://doi.org/10.1021/acs.analchem.6b02588.
